# PINOT NOIR: Pulmonic INsufficiency imprOvemenT with Nitric Oxide Inhalational Response

**DOI:** 10.1186/1532-429X-14-S1-O73

**Published:** 2012-02-01

**Authors:** Stephen Hart, Ganesh Devendra, Yuli Y Kim, Scott D Flamm, Sagar Kalahasti, Janine Arruda, Esteban Walker, Thananya Boonyasiranant, Michael Bolen, Randolph M Setser, Richard Krasuski

**Affiliations:** 1 Cardiovascular Imaging Laboratory, Cleveland Clinic, Cleveland, OH, USA; 2 Lerner College of Medicine of Case Western Reserve University, Cleveland Clinic, Cleveland, OH, USA; 3 Pediatric Cardiology, Cleveland Clinic, Cleveland, OH, USA; 4 Quantitative Health Sciences, Cleveland Clinic, Cleveland, OH, USA; 5 Cardiovascular Medicine, Cleveland Clinic, Cleveland, OH, USA; 6 Hospital of the University of Pennsylvania and Children’s Hospital of Philadelphia, University of Pennsylvania, Philadelphia, PA, USA

## Background

Tetralogy of Fallot (TOF) repair and pulmonary valvotomy for pulmonary stenosis (PS) often lead to progressive pulmonary insufficiency (PI), right ventricular enlargement and dysfunction. This study assessed whether the pulmonary regurgitant fraction measured by cardiac magnetic resonance imaging (CMR) could be reduced by a selective pulmonary vasodilator.

## Methods

Patients with at least moderate PI by echocardiography undergoing a clinically indicated CMR study were prospectively enrolled. Patients with a RV-PA conduit or residual pulmonic stenosis were excluded. Ventricular volume and blood flow sequences were obtained at baseline and after administration of 40ppm inhaled nitric oxide (iNO). Eleven Sixteen patients (11 with repaired TOF and 5 with repaired PS) completed the protocol with adequate data for analysis.

## Results

The median age [range] was 35 [19 - 46] years, BMI was 26±5 kg/m^2^ (mean ± standard deviation), 50% were women and 75% were in NYHA class I. Right ventricular end diastolic volume index for the cohort was 157±33 mL/m^2^, end systolic volume index was 93±20 mL/m^2^ and right ventricular ejection fraction was 40±6%. Baseline pulmonary regurgitant volume was 45±25 mL/beat and regurgitant fraction was 35±16%. During administration of iNO, regurgitant volume was reduced by an average of 6±9% (p=0.01) and regurgitant fraction was reduced by an average of 5±8% (p=0.02). No statistically significant changes were observed in stroke volume, ejection fraction or cardiac output for either the left or right ventricle.

## Conclusions

iNO administration appears to reduce the regurgitant fraction in patients with at least moderate PI suggesting a potential role for selective pulmonary vasodilator therapy in these patients.

## Funding

Cleveland Clinic Imaging Institute Research Council.

**Figure 1:**
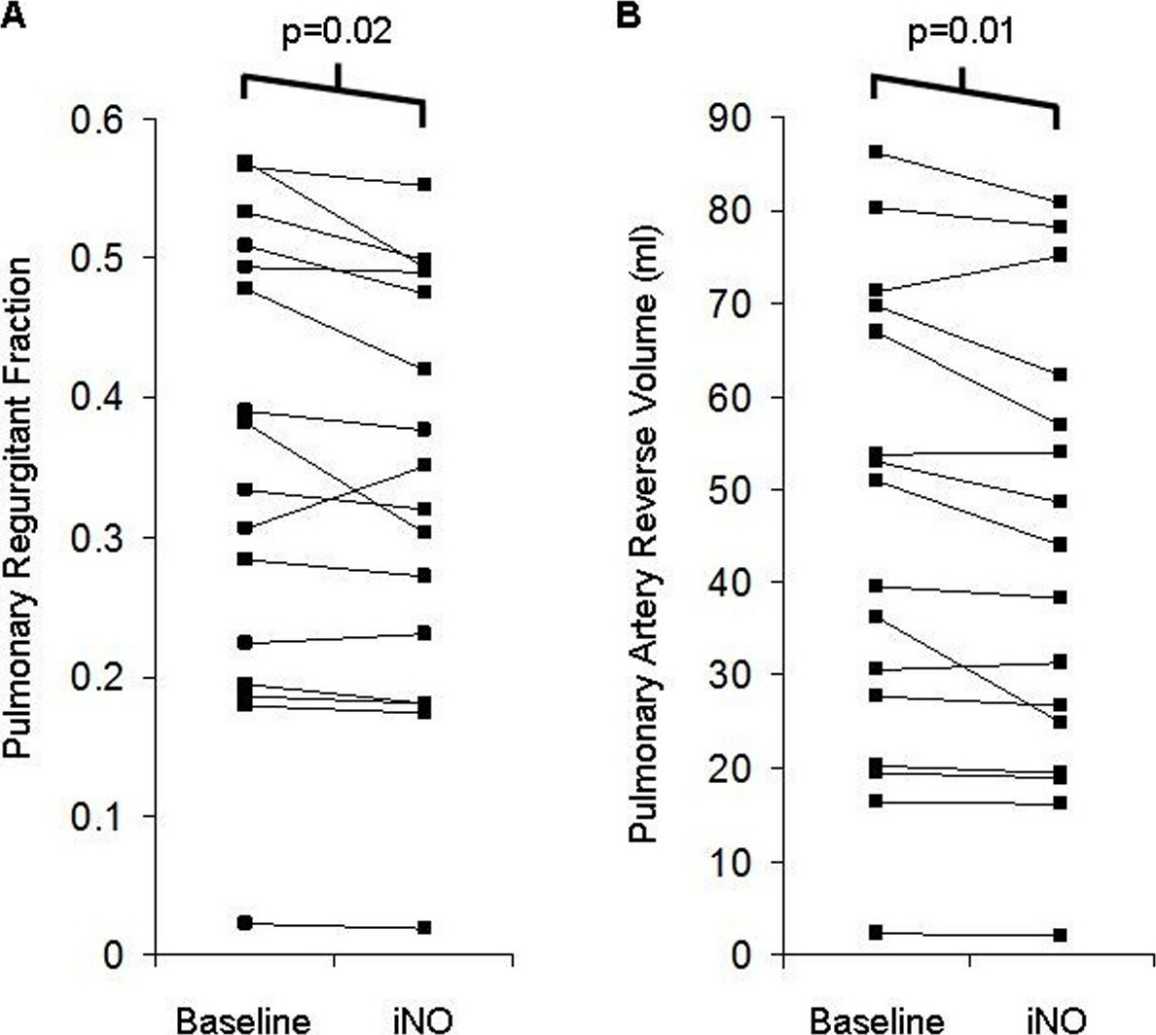
Effect of iNO on pulmonary insufficiency (A) and on pulmonary artery reverse volume (B) (n=16).

